# Diversity and antibacterial activity of fungal endophytes from *Eucalyptus exserta*

**DOI:** 10.1186/s12866-021-02229-8

**Published:** 2021-05-27

**Authors:** Ziling Mao, Weihao Zhang, Chunyin Wu, Hao Feng, Yuanhang Peng, Hamza Shahid, Zining Cui, Ping Ding, Tijiang Shan

**Affiliations:** 1 Guangdong Key Laboratory for Innovative Development and Utilization of Forest Plant Germplasm, College of Forestry and Landscape Architecture, South China Agricultural University, 510642, No. 483, Wushan Road, Tianhe District, Guangdong 510642 Guangzhou, China; 2 Guangdong Province Key Laboratory of Microbial Signals and Disease Control, South China Agricultural University, 510642 Guangzhou, China; 3 School of Pharmaceutical Sciences, Guangzhou University of Chinese Medicine, No. 232, Waihuandong Road, Panyu District, Guangdong 510006 Guangzhou, China

**Keywords:** Endophytic fungi, *Eucalyptus exserta*, Secondary metabolites, Antibacterial activity, *Lophiostoma* sp. Eef-7, *Ralstonia solanacearum*

## Abstract

**Background:**

Eucalyptus bacterial wilt caused by *Ralstonia solanacearum* is an important eucalyptus disease. Endophytic fungi, an important source of natural active substances, provide a new breakthrough for the control of plant diseases.

**Results:**

In the present study, 80 endophytic fungal isolates were obtained from the healthy branches and fruits of *Eucalyptus exserta*. Fifteen distinct isolates (MK120854-MK120868) were selected for further taxonomic identification through morphological trait assessments and internal transcribed spacer (ITS) region-rRNA gene sequence analysis. Thirteen genera, namely, *Phyllosticta*, *Penicillium*, *Eutypella*, *Purpureocillium*, *Talaromyces*, *Lophiostoma*, *Cladosporium*, *Pestalotiopsis*, *Chaetomium*, *Fusarium*, *Gongronella*, *Scedosporium* and *Pseudallescheria*, were identified on the basis of their morphological characteristics. Members of the genus *Phyllosticta* were the primary isolates, with a colonization frequency (CF) of 27.5 %. Most of the fungal isolates displayed antibacterial activity. The crude extracts obtained from *Lophiostoma* sp. Eef-7, *Pestalotiopsis* sp. Eef-9 and *Chaetomium* sp. Eef-10 exhibited strong inhibition on the test bacteria, and *Lophiostoma* sp. Eef-7 was further cultured on a large scale. Three known compounds, scorpinone (1), 5-deoxybostrycoidin (2) and 4-methyl-5,6-dihydro-2* H*-pyran-2-one (3), were isolated from the endophytic fungus *Lophiostoma* sp. Eef-7 associated with *E. exserta*. The structures of these compounds were elucidated by analysis of 1D and 2D NMR and HR-ESI-MS spectra and a comparison of their spectral data with published values. Compounds 1 and 2 showed weak antimicrobial activity against *Ralstonia solanacearum*.

**Conclusions:**

Endophytic fungi from *Eucalyptus exserta* may represent alternative sources of antimicrobial agents. *Lophiostoma* sp. Eef-7 can produce 2-azaanthraquinone derivatives and shows weak antibacterial activity against *Ralstonia solanacearum*.

**Supplementary Information:**

The online version contains supplementary material available at 10.1186/s12866-021-02229-8.

## Background

Eucalyptus bacterial wilt caused by *Ralstonia solanacearum* (Smith) Yabuuchi is a destructive, systemic vascular bundle disease referred to as cancer in eucalyptus. In recent years, this disease has been prevalent in South China and has become a major obstacle to the development of eucalyptus forests, severely threatening the sustainable development of the eucalyptus industry [1]. Plant diseases caused by pathogenic bacteria directly result in enormous losses in the agricultural economy each year and are significant issues that need to be resolved [2, 3]. Currently, chemical pesticides such as antibiotics are some of the most efficient and cost-effective methods of controlling plant pathogenic bacteria. However, with the long-term use of pesticides in large quantities, the emergence of drug-resistant pathogenic bacterial strains has made this problem even more intractable [4]. In recent years, the extensive and frequently inappropriate use of antibiotics has caused pathogenic bacteria to develop resistance to commercial drugs. Consequently, the search for antibacterial substances with novel structures and outstanding bioactivity from natural products and developing them into commercial pesticides has become a research hotspot in agriculture [5].

Natural products such as medicines have likely been used by humans for thousands of years. These products are derived from natural sources such as microorganisms, plants or animals [6]. Plant endophytes are microorganisms that reside in the tissues of living plants without causing any apparent adverse effects to their host plants [7–9]. Endophytic fungi of plants are known as a feracious source of natural products with interesting bioactivities, such as antibacterial, larvicidal, antioxidant, anti-inflammatory, and phytotoxic activities [10–14]. In the past few years, an increasing number of active substances with unique chemical structures have been isolated from endophytic fungi using a combination of morphological, phylogenetic and metabolomics analyses [15, 16]. From 1981 to 2010, more than 50 % of small molecules originated from natural products [17]. The main antibacterial, antifungal, antiviral and anticancer compounds came from natural sources such as plants, fungi, and even bacteria. These compounds have been isolated from previously studied plants, including bryophytes [18], pteridophytes [19], herbage [20] and angiosperms [21]. Most endophytic fungi produce bioactive secondary metabolites such as alkaloids, terpenoids, steroids, quinones, isocoumarins, lignans, phenylpropanoids, phenols, and lactones [22–24].

*Eucalyptus exserta* is a well-known Chinese afforestation tree species that is primarily distributed in South China and has leaves that produce essential oil with antimicrobial activities. To date, few studies have investigated endophytic fungi from *E. exserta*. As part of our ongoing investigation of endophytic fungi from *E. exserta*, which has been reported to have endophytes with antimicrobial activity, attracted our attention [25]. Thus, the goal of this present study was to isolate and identify endophytic fungi from *E. exserta* and to search for endophytic fungi capable of producing bioactive substances with antimicrobial activity. The isolation, structural elucidation, and antimicrobial activities of metabolites from a terrestrial *Lophiostoma* strain are reported for the first time.

## Results

### Identification of the endophytic fungi

**Fig. 1:**
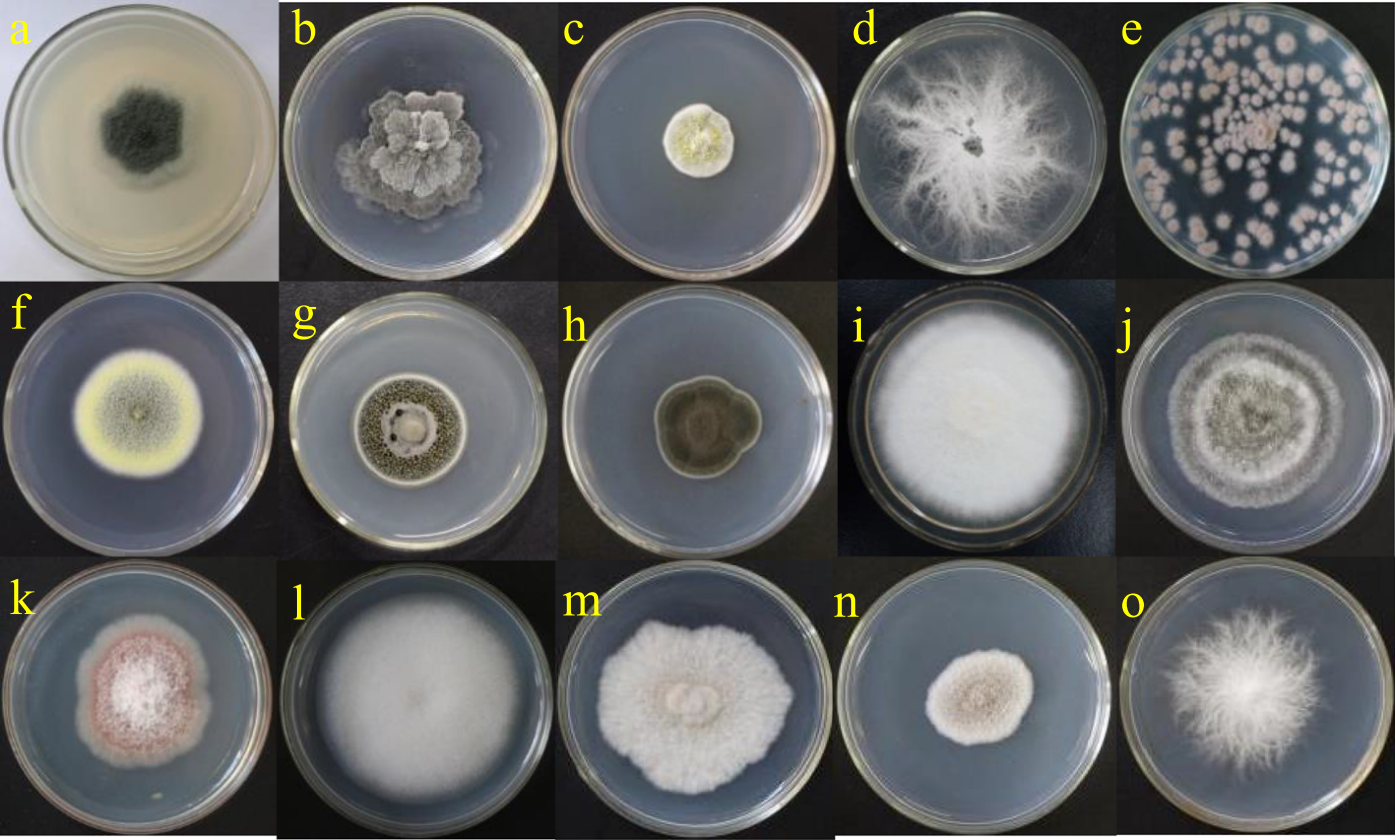
Endophytic Fungi isolated from *Eucalyptus exserta*. a~o were endophytic fungal isolates Eef-1~Eef-15, respectively

In the present study, 80 fungal isolates were obtained from healthy branches and fruits of *E. exserta*. According to their morphological features, the isolates were preliminarily grouped through dereplication into fifteen different morphological taxa (Fig. 1) that were then selected for DNA sequence analysis using the ITS region. The ITS (ITS4-5.8 S-ITS5) sequences of these endophytic fungi were compared to corresponding reference fungal taxa sequences in GenBank. Based on the macro- and microscopic identification results, they were identified as members of thirteen genera, including *Phyllosticta*, *Penicillium*, *Eutypella*, *Purpureocillium*, *Talaromyces*, *Lophiostoma*, *Cladosporium*, *Pestalotiopsis*, *Chaetomium*, *Fusarium*, *Gongronella*, *Scedosporium* and *Pseudallescheria* (Table 1). The diversity of these fungi associated with *E. exserta* was revealed by evaluating their colonization frequency (CF). The fungi of the genus *Phyllosticta* were the primary isolates, with a CF of 27.5 %, followed by those of the genus *Eutypella*, with a CF of 10 %. The ITS4-5.8 S-ITS5 partial sequences of 15 isolates were submitted to GenBank to obtain accession numbers (MK120854~MK120868), and the closest related species were obtained by BLAST analysis (Table 1). The results showed that all the sequences had more than 98 % similarity with the species in GenBank.

**Table 1:** Identification of endophytic fungi isolated from *Eucalyptus exserta*

Fungal isolate	CF (%)	Accession number	Macro- and microscopic identification	Closest raleted species	Similarity (%)
Eef-1	12.50(10/80)	MK120854	*Phyllosticta* sp.	EU167584.1 *Phyllosticta elongata*	100
Eef-2	15.00(12/80)	MK120855	*Phyllosticta* sp.	EU167584.1 *Phyllosticta elongata*	99
Eef-3	5.00(4/80)	MK120856	*Penicillium* sp.	NR153252.1 *Penicillium citreosulfuratum*	99
Eef-4	2.50(2/80)	MK120857	*Eutypella* sp.	KY962999.1 *Eutypella scoparia*	98
Eef-5	6.25(5/80)	MK120858	*Purpureocillium* sp.	KC157751.1 *Purpureocillium lilacinum*	100
Eef-6	7.50(6/80)	MK120859	*Talaromyces* sp.	MF093899.1 *Talaromyces pinophilus*	99
Eef-7	2.50(2/80)	MK120860	*Lophiostoma* sp.	HQ914838.1 *Lophiostoma* sp.	98
Eef-8	2.50(2/80)	MK120861	*Cladosporium* sp.	MF473305.1 *Cladosporium tenuissimum*	100
Eef-9	5.25(5/80)	MK120862	*Pestalotiopsis* sp.	LC184194.1 *Pestalotiopsis* sp.	99
Eef-10	5.00(4/80)	MK120863	*Chaetomium* sp.	KU504292.1 *Chaetomium* sp.	100
Eef-11	7.50(6/80)	MK120864	*Fusarium* sp.	LT841250.1 *Fusarium proliferatum*	99
Eef-12	7.50(6/80)	MK120865	*Gongronella* sp.	KM246758.1 *Gongronella butler*i	99
Eef-13	3.75(3/80)	MK120866	*Scedosporium* sp.	KC202949.1 *Scedosporium boydii*	99
Eef-14	5.00(4/80)	MK120867	*Pseudallescheria* sp.	KP132615.1 *Pseudallescheria angusta*	99
Eef-15	7.50(6/80)	MK120868	*Eutypella* sp.	KT868952.1 *Eutypella* sp.	99

### Phylogenetic relationship analysis of the fungal endophytes

**Fig. 2:**
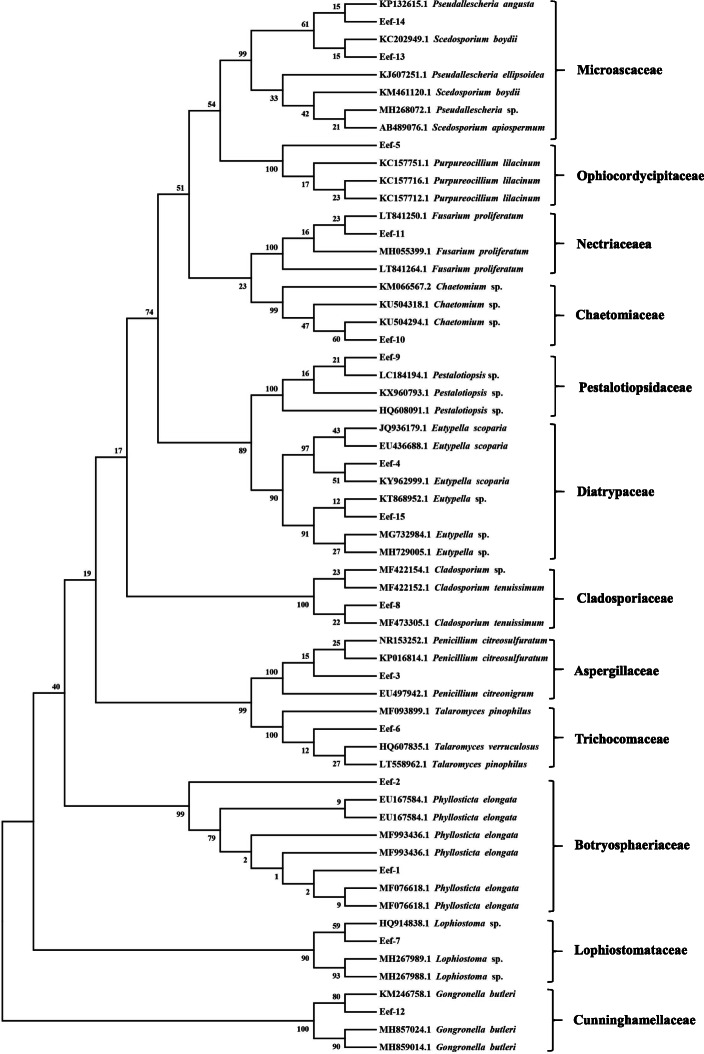
Phylogenetic tree of endophytic fungi isolated from *E. exserta* based on the rDNA-ITS sequence.  The tree was constructed with the maximum likelihood method in MEGA 6.0 using default parameters, and bootstrap values were calculated after 1,000 replications.

The ITS sequence data of fifteen fungal endophytes from *E. exserta* were submitted to GenBank under the accession numbers MK120854 to MK120868 (Table 1), and sequence data of the closest related species were obtained from the same database. These datasets were used to construct a phylogenetic tree with the maximum likelihood method and analyze the phylogenetic affiliations of these endophytes (Fig. 2). The 15 fungal isolates could be sorted into 12 groups, Microascaceae, Ophiocordycipitaceae, Nectriaceae, Chaetomiaceae, Pestalotiopsidaceae, Diatrypaceae, Cladosporiaceae, Aspergillaceae, Trichocomaceae, Botryosphaeriaceae, Lophiostomataceae and Cunninghamellaceae, all of which belong to Ascomycota except for isolate Eef-12 (*Gongronella* sp.), which belongs to the phylum Zygomycota.

### Antimicrobial activity screening of EtOAc extracts from endophytic fungi

The antibacterial activity results of the ethyl acetate (EtOAc) extracts from the endophytic fungi against seven test bacteria using a thin layer chromatography (TLC)-bioautography assay are shown in Table 2. The results showed that most of the EtOAc extracts displayed specific inhibitory activity, with a range of inhibitory activity observed against different tested bacteria. Some fungal isolates (e.g., Eef-6, Eef-9, Eef-10, Eef-11 and Eef-12) displayed antibacterial activities against all tested bacteria. Eef-4 displayed antibacterial activities against six tested bacteria except for *Xanthomonas vesicatoria*. The EtOAc extracts of the endophytic fungi Eef-2, Eef-7, Eef-13 and Eef-14 showed inhibitory activities against five tested bacteria, while Eef-3, Eef-5 and Eef-15 showed weak inhibition and displayed antibacterial activities against four tested bacteria.

Interestingly, thirteen isolates showed antibacterial activity against *R. solanacearum*. Among these isolates, Eef-9 and Eef-10 displayed the best inhibition, with inhibition spot diameters greater than 10 mm for both strains, while those of the remaining isolates were all less than 10 mm. In addition, the endophytic fungus Eef-9 also displayed the best antibacterial activity against other tested bacteria, with inhibition spot diameters greater than 10 mm for all bacterial strains. The inhibition spot diameters of the endophytic fungus Eef-10 against *Staphylococcus haemolyticus*, *X. vesicatoria* and *Pseudomonas lachrymans* were more than 10 mm. The endophytic fungus Eef-7 did not show inhibitory activity against *X. vesicatoria* and *Escherichia coli*, but the inhibition spot diameters against *Bacillus subtilis* and *S. haemolyticus* were more than 10 mm. In addition, although Eef-6, Eef-11 and Eef-12 inhibited all tested bacteria, the inhibition spot diameters were less than 10 mm.

The *R*_f_ value is primarily related to the polarity of the secondary metabolites produced by endophytic fungi, where larger *R*_f_ values indicate lower polarity. In addition, a range of observed *R*_f_ values is related to the number and antibacterial activity of the secondary metabolites, with a larger range of the *R*_f_ values indicating that more antibacterial compounds have stronger antibacterial activities. The results showed that Eef-9, Eef-10 and Eef-7 displayed strong antibacterial activities and had the ability to produce valuable compounds. Therefore, this three endophytic fungi could be selected for further analysis and used as candidate strains for the further isolation and identification of active components.

**Table 2:** Antibacterial activities of crude extracts of endophytic fungi isolated from *E. exserta*

Strains	*R*_f_ value (Inhibition spot diameter)						
	*R. solanacearum*	*S. aureus*	*B. subtilis*	*S. haemolyticus*	*X. vesicatoria*	*P. lachrymans*	*E. coli*
Eef-1	0.04–0.10^+^	0.00-0.28^++^	0.00-0.19^++^	---	0.00-0.42^+^	---	---
Eef-2	0.00-0.49^++^	0.00-0.38^++^	0.00-0.27^++^	---	0.00-0.38^+^; 0.48–0.60^++^	0.02–0.03^+^; 0.08–0.11^+^; 0.24–0.27^+^; 0.37–0.44^+^	---
Eef-3	0.00-0.38^+^; 0.47–0.52^+^	0.15–0.75^++^	---	---	0.00-0.70^++^	---	0.52–0.55^+^
Eef-4	0.34–0.52^++^	0.00-0.09^+^; 0.12–0.97^++^	0.03–0.06^+^; 0.16–0.26^+^; 0.32–0.57^++^	0.11–0.22^+^; 0.42–0.83^++^	---	0.06–0.43^+^; 0.55–0.63^++^	0.00-0.11^+^; 0.25–0.35^++^
Eef-5	0.00-0.27^+^; 0.32–0.37^+^; 0.52–0.71^+^	0.00-0.62^+^	0.00-0.73^++^	---	---	0.19–0.37^+^; 0.60–0.70^+^	---
Eef-6	0.00-0.66^++^	0.00-0.22^++^	0.00-0.22^++^	0.14–0.17^+^	0.00-0.34^++^; 0.35–0.42^++^	0.17–0.30^+^	0.20–0.34^+^; 0.55–0.73^+^
Eef-7	0.03–0.17^++^	0.03–0.86^++^	0.03–0.19^++^; 0.28–0.48^++^; 0.49–0.93^+++^	0.17–0.29^++^; 0.49–0.95^+++^	---	0.00-0.82^++^	---
Eef-8	0.12–0.18^+^; 0.38–0.62^+^; 0.78–0.86^++^	0.15–0.75^++^	0.19–0.48^++^	---	---	0.03–0.29^+^; 0.68–0.72^+^	---
Eef-9	0.00-0.31^+^; 0.41–0.49^+++^	0.00-0.23^+++^	0.00-0.28^+++^	0.0-0.20^++^; 0.67–0.70^+++^	0.00-0.29^+++^	0.00-0.24^+^; 0.32–0.49^++^; 0.57–0.65^+++^	0.00-0.18^+++^
Eef-10	0.00-0.78^+++^	0.05–0.25^++^	0.06–0.17^+^; 0.23–0.36^++^	0.0-0.92^+++^	0.00-0.82^+++^	0.00-0.81^+++^	0.00-0.72^+^; 0.82–0.91^++^
Eef-11	0.00-0.07^+^	0.00-0.17^++^; 0.22–0.27^+^; 0.37–0.42^++^	0.00-0.08^+^; 0.13–0.17^+^; 0.35–0.48^+^	0.00-0.27^++^	0.00-0.17^++^; 0.22–0.28^+^; 0.33–0.43^++^;	0.20–0.25^+^; 0.35–0.4^+^	0.10–0.18^+^
Eef-12	0.00-0.05^+^	0.00-0.17^++^; 0.20–0.23^+^	0.00-0.18^++^; 0.18–0.25^+^	0.00-0.5^++^	0.00-0.4^++^	0.00-0.08^++^	0.00-0.20^++^
Eef-13	---	0.00-0.05^+^; 0.38–0.47^+^	0.13–0.32^+^; 0.38–0.52^++^	0.00-0.08^+^	0.00-0.38^++^	0.00-0.2^++^; 0.30–0.45^++^	---
Eef-14	---	0.00-0.22^++^	0.00-0.22^++^	---	0.00-0.34^+^; 0.35–0.42^++^	0.17–0.30^+^	0.20–0.34^+^; 0.55–0.73^+^
Eef-15	0.00-0.38^+^; 0.47–0.52^+^	0.15–0.75^++^	---	---	0.00-0.70^++^	---	0.52–0.55^+^
Streptomycin sulfate	++	++	+++	++	++	+++	++

---: Inhibition spot was not observed; +: Maximum inhibition spot diameter d <5 mm; ++: Maximum inhibition spot diameter 5 mm ≤ d <10 mm; +++: Maximum inhibition spot diameter d ≥ 10 mm; The positive control streptomycin sulfate was only sampled on TLC plate.

### Purification and structure elucidation

**Fig. 3:**
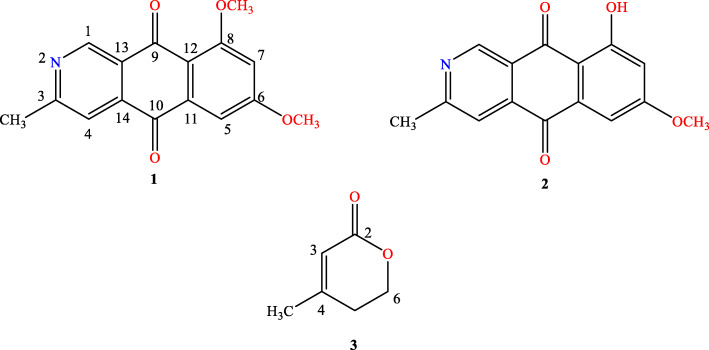
Chemical structures of scorpinone (1), 5-​deoxybostrycoidin (2) and 4-methyl-5,6-dihydro-2* H*-pyran-2-one (3)

Eef-7 was selected for large-scale fermentation using sterilized rice medium. The solid fermentation product of the Eef-7 was extracted with methanol (MeOH), and the resulting extracts were partitioned into petroleum ether (PE)- and EtOAc-soluble fractions. The PE and EtOAc fractions were further purified by conventional chromatographic techniques, and three known compounds, scorpinone (**1**), 5-​deoxybostrycoidin (**2**) and 4-methyl-5,6-dihydro-2* H*-pyran-2-one (**3**), were structurally characterized (Fig. 3).

The chemical structures of compounds 1–2 were elucidated through HR-ESI-MS and 1D and 2D NMR experiments (COSY, HSQC and HMBC), and compound 3 was elucidated according to the ^1^ H NMR and ^13^ C NMR data and compared with the literature (Figs. S1, S2, S3, S4, S5, S6, S7, S8, S9, S10, S11, S12, S13, S14).

**Fig. 4:**
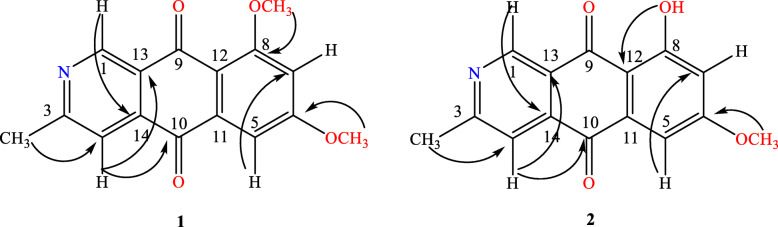
Key HMBC (H-C) correlations of scorpinone (**1**) and 5-​deoxybostrycoidin (**2**)

Scorpinone (**1**): yellow crystal; HR-ESI-MS m/z 284.092445 [M + H]^+^ (calcd. for C_16_H_14_NO_4_, 284.09228), 306.074175 [M + Na]^+^ (calcd. for C_16_H_13_NNaO_4_, 306.07423); ^1^ H NMR and ^13^ C NMR see Table 3; and the key HMBC correlations see Fig. 4. The structure of compound **1** was confirmed by comparison with literature data [26].

5-​Deoxybostrycoidin (**2**): light yellow power; HR-ESI-MS m/z 268.061917 [M-H]^−^ (calcd. for C_15_H_10_NO_4_, 268.06098); ^1^ H NMR and ^13^ C NMR see Table 3; and the key HMBC correlations see Fig. 4. The data were consistent with the literature [27].

**Table 3:** ^13^ C-NMR and ^1^ H-NMR data for compounds 1–2 (in CDCl_3_)

Position	1(*δ* in ppm, *J* in Hz)		2 (*δ* in ppm, *J* in Hz)	
	*δ*_C_	*δ*_H_	*δ*_C_	*δ*_H_
1	149.89	9.40 (s)	149.20	9.41 (s)
3	162.94	-	165.69	-
3-CH_3_	25.17	2.74 (s)	25.43	2.77 (s)
4	117.57	7.80 (s)	118.67	7.86 (s)
5	103.74	7.42 (d, 2.4)	107.53	7.34 (d, 2.5)
6	164.26	-	166.01	-
6- OCH_3_	56.49	4.01 (s)	56.28	3.94 (s)
7	105.64	6.83 (d, 2.4)	108.34	6.74 (d, 2.5)
8	165.14	-	166.58	-
8-OH	-	-	-	12.76 (s)
8-OCH_3_	56.75	3.99 (s)	-	-
9	180.61	-	182.42	-
10	183.64	-	186.31	-
11	137.16	-	138.74	-
12	115.87	-	110.56	-
13	137.70	--	124.28	-
14	125.62		134.61	-

4-Methyl-5,6-dihydro-2* H*-pyran-2-one (**3**): colorless oil; ^1^ H NMR (acetone-*d*_6_, 600 MHz) *δ*_H_ 5.71(1 H, s, H-3), 4.32 (2 H, t, *J* = 6.2 Hz, H-6), 2.42 (2 H, t, *J* = 6.2 Hz, H-5), 2.01(3 H, s, H-7); and ^13^ C NMR (acetone-*d*_6_, 151 MHz) *δ*_C_ 164.55 (C-2), 159.51 (C-4), 116.95 (C-3), 66.64 (C-6), 29.75 (C-5), 22.87 (C-7). The ^1^ H NMR and ^13^ C NMR data were consistent with the literature [28].

### Antibacterial activities of pure compounds against ***R. solanacearum***

**Table 4:** Antibacterial activities of compounds 1 and 2 against *R. solanacearum*

Samples	Inhibition zone diameter (mm)				
	4 µg	8 µg	16 µg	32 µg	64 µg
1	6.43 ± 0.69	8.71 ± 0.53	8.98 ± 0.71	9.33 ± 0.63	9.86 ± 0.75
2	7.19 ± 0.22	8.04 ± 0.39	8.71 ± 0.30	9.12 ± 0.22	9.58 ± 0.60

Compounds 1 and 2 were further evaluated for their antibacterial activities against *R. solanacearum*. Both compounds 1 and 2 showed weak antibacterial effects against the tested bacterium (Table 4). The inhibition zone diameter of streptomycin sulfate was 13.03 ± 0.43 mm at an additive amount of 6.25 µg.

## Discussion

Endophytic fungi that colonize plant tissues cause no symptoms of tissue damage and play important roles in the ecosystem function of their hosts [29, 30]. Endophytic fungi have formed a symbiotic relationship with their host plants in the long-term process of coevolution, providing nutrients that promote the defense mechanisms of plants and enhancing their ability to resist adverse conditions, diseases, and insect pests [31, 32]. Endophytic fungi are widely present in all parts of plants, but the species and quantity of endophytic fungi vary greatly in different plants and in different parts of the same plant [1]. Endophytic fungal species are also related to the growing age, season and different tissues and organs of the host plant [33]. In the present study, only 15 different endophytic fungi were isolated and identified from the branches and fruits of *E. exserta*, a result that may be related to the host plants and their living environment. Most endophytic fungi cannot be cultured on artificial medium, and as potato dextrose agar (PDA) is not necessarily an optimal medium, the 15 endophytic fungi isolated in this study represent only some of the endophytic fungi of *E. exserta*. In subsequent studies, different media and high-throughput sequencing can be used to isolate as many endophytic fungi as possible, and the distribution and growth of endophytic fungi in different media can be compared.

Endophytic fungi play a crucial role in the agricultural and forestry sectors owing to their ability to improve crop yield, enhance plant disease resistance and reduce the use of chemical pesticides, which is beneficial for the protection of the environment and human health [34]. Some bioactive secondary metabolites isolated from endophytic fungi have shown significant economic value and application prospects in the biological control of plant diseases and in drug research and development [35]. Endophytic fungi are a reservoir of antibacterial agents [24]. For example, the endophytic fungus *Nigrospora sphaerica* was isolated from the leaves of *Indigofera suffruticosa*, which has been shown to produce bioactive agents with pharmaceutical potential [22]. Nine new Spirobisnaphthalenes were isolated from the endophytic fungi *Berkleasmium* sp. associated with *Dioscorea zingiberensis*, and all isolated compounds have been evaluated for their antibacterial activities against six different bacteria [36]. The isolation and identification of endophytic mycobiota is necessary since the medicinal properties of a plant can result from the ability of its endophytic microorganisms to produce biologically active secondary metabolites [37, 38]. In the present study, the endophytic fungi Eef-9 (*Pestalotiopsis* sp., MK120862), Eef-10 (*Chaetomium* sp., MK120863) and Eef-7 (*Lophiostoma* sp., MK120860) displayed potent antibacterial activities and had the ability to produce valuable compounds that require further analysis. At present, strains of the same genus as Eef-9 (*Pestalotiopsis* sp., MK120862), Eef-10 (*Chaetomium* sp., MK120863) and Eef-7 (*Lophiostoma* sp., MK120860) have been reported as endophytic fungi in a variety of plants. Pestalotiopisorin B, a new isocoumarin derivative isolated from the mangrove endophytic fungus *Pestalotiopsis* sp. HHL101, exhibited modest antibacterial activity against *Escherichia coli* and *Pseudomonas aeruginosa* [39]. Two new butenolides (pestalolides B and C) and two new diphenyl ethers (pestalotethers E and F) together with seven known compounds were isolated from the endophytic fungus *Pestalotiopsis* sp. living in the leaves of tea trees and displayed cytotoxic activities against four different human tumor cell lines [40]. In our previous study, seven depsidones, including four new depsidones mollicellins O-R, were isolated from cultures of the endophytic fungus *Chaetomium* sp. Eef-10. Mollicellin H displayed the best antibacterial activity against *S. aureus* ATCC29213 and *S. aureus* N50 (MRSA), and mollicellin G was active against two human cancer cell lines (HepG2 and HeLa). In addition, mollicellin O showed antioxidant activity with an IC_50_ value of 71.92 µg/mL [7].

Although members of the genus *Lophiostoma* have been reported as endophytes or saprophytes from freshwater, terrestrial or marine environments, registered chemical investigations have primarily focused on marine-derived species [29, 41, 42]. Lophiostomin A-D, new 3,4-dihydroisocoumarin congeners, were isolated from the endophytic fungus *Lophiostoma* sp. of *Siraitia grosvenorii*, and lophiostomin A and B displayed moderate inhibitory activities against the germination of *Magnaporthe oryzae* spores [29]. In the present study, three known compounds were isolated from the endophytic fungus *Lophiostoma* sp. Eef-7, and compounds 1 and 2 were both 2-azaanthraquinones, a class of polyketide-derived heterocycles primarily produced in nature by fungi or lichens that display phytotoxic effects and activity against various microorganisms [43, 44]. Scorpinone (1) has been isolated from a newly described fungus, *Amorosia littoralis* [26], and the biogenetic origin of the carbon atoms was assessed through isotopic enrichment studies using [2-^13^ C]-acetate and [1,2-^13^ C]-acetate. The labeling results revealed that a heptaketide precursor is involved in the biosynthesis of scorpinone (**1**), which as has also been observed for the structurally related naphthoquinone dihydrofusarubin [44]. Compound 2 was reported as a new melanin, a secondary metabolite made up of complex heterogeneous polymers of phenolic and/or indolic monomers [45]. 5-​Deoxybostrycoidin (2) is located within the periderm of perithecia of the *Fusarium graminearum* group and synthesized from the reaction of anhydrofusarubin derivatives and ammonia [46]. Several *Fusarium* species, including *F. graminearum*, *F. verticillioides*, and *F. fujikuroi* [46, 47], produce blackish perithecial pigments. The fungus *Nectria haematococca* has also been reported to produce compound 2 [27], a pigment that is important for UV or desiccation protection during the differentiation of perithecia and ascospores [48–50]. Compound 2 also displayed significant cytotoxicity against the MDA-MB-435 and NCI-H460 cell lines, with IC_50_ values of 5.32 and 6.57 µM, respectively [51].

## Conclusions

In the present study, endophytic fungi were reported from the Chinese afforestation tree species *E. exserta* for the first time and exhibited antibacterial activity based on TLC bioautography assays. Fifteen endophytic fungi were identified from *E. exserta*, and Eef-9, Eef-10 and Eef-7 displayed potent antibacterial activities. All strains showed antibacterial activity, including four with wide-spectrum activity. The percentage of endophytic fungi isolated from fruits and branches of *E. exserta* revealed the enormous capacity for the bioactive compounds production with antimicrobial potential. The three most active strains (*Pestalotiopsis* sp. Eef-9, *Chaetomium* sp. Eef-10 and *Lophiostoma* sp. Eef-7) will be studied for taxonomy and isolation of antimicrobial compounds by using liquid and semisolid fermentation assays. In summary, endophytic fungi from *E. exserta* contribute to antimicrobial activity of the host plant and should be investigated as alternative sources of antimicrobial agents in the future.

## Materials and methods

### Plant material

Healthy branches and fruits of *Eucalyptus exserta* F. V. Muell. were collected in June 2014 from the campus of South China Agricultural University (SCAU, 23°10’ N, 113°21’ E at an altitude of 23.6 m), Guangzhou, Guangdong Province, China. Guangzhou is located on the subtropical coast, and the Tropic of Cancer runs through the central and southern parts of the city. This region has a maritime subtropical monsoon climate, characterized by warm and rainy conditions, sufficient light and heat, long summers and short frost periods. The annual average temperature ranges from 20 to 22 °C, and the average annual rainfall ranges from 1,623.6-1,899.8 mm. The taxonomic identification of the plant materials was performed by Dr. Mingxuan Zheng of College of Forestry and Landscape Architecture (SCAU), where the voucher specimen (SCAULPMH-140,605) of the plant was deposited.

### Isolation and cultivation of endophytic fungi

The isolation of endophytic fungi was performed by following the process described in our previous report with some modifications [20]. Briefly, first, the healthy branches and fruits of *E. exserta* collected from eight different trees were thoroughly washed under running tap water. Then, the samples were surface sterilized by dipping them in 75 % ethanol for 30 s, followed by immersion in 0.2 % mercuric chloride for 20 min. The samples were then rinsed in sterile distilled water three times (5 min each time) and finally dried on sterile filter paper. The epidermis of each branch explant was removed with a sterile scalpel. The sterilization process was confirmed by placing the sterile epidermal tissues on PDA (200 g/L potato, 20 g/L dextrose, and 20 g/L agar) Petri dishes. After sterilization, each explant (without epidermis) was cut into 5 × 5 × 5 mm cubes, which were individually placed on PDA plates supplemented with streptomycin sulfate (500 mg/L) to suppress bacterial growth. After the plates were incubated in the dark at 28 °C for 7–30 days, the number of fungi was counted, and each fungal colony was isolated and subcultured to obtain a pure culture. The CF of each endophyte was also calculated [52]. CF (%) = (*N*_*COL*_/*N*_*t*_) × 100, where *N*_*COL*_ is the number of cubes colonized by each fungus and *N*_*t*_ is the total number of cubes. All the isolated fungi were deposited at the College of Forestry and Landscape Architecture, South China Agricultural University.

### General experimental procedures

The morphological characteristics of the endophytic fungi isolated from *E. exserta* were observed using a CX31 Digital Fluorescence Microscope (Mshot, Guangzhou, China). High-resolution electrospray ionization mass spectrometry (HR-ESI-MS) was carried out on a Q-TOF mass spectrometer from Bruker maXis with an ESI interface (Bruker, Fremont, CA, USA). Nuclear magnetic resonance (NMR; ^1^ H and ^13^ C) spectra were recorded on a Bruker Avance-600 NMR spectrometer (^1^ H at 600 MHz and ^13^ C at 151 MHz) (Bruker, Fremont, CA, USA). Semi-preparative HPLC separation was performed on a Lumtech instrument equipped with an HPLC K-501 pump and a K-2501 UV detector using a Wondasil 38020-41 C_18_ column (250 mm × 21.2 mm, 5 μm, Welch Materials Inc.). Water used for experiments was purified with an ultrapure water machine (Exceed-Cb-10, Aike, Chengdu, China). MeOH was of HPLC grade, and all the other reagents were of analytical grade. All solvents used for HPLC were filtered through a 0.45 μm nylon membrane before use. Silica gel (60–100, 100–200 and 200–300 mesh, Qingdao Marine Chemical Inc.) and Sephadex LH-20 (GE Healthcare) were used for separation and isolation. Precoated silica gel GF-254 plates (Qingdao Marine Chemical Inc.) were used for analytical TLC. Spots were visualized under UV light (254 or 356 nm).

### Morphological characterization

The morphological characteristics, including colony diameter, texture, color, dimensions and morphology of hyphae and conidia of the fungal isolates, were observed and described according to previously described methods [52].

### DNA extraction, ITS-rDNA amplification and sequence analysis

Total genomic DNA of the fungal isolates was prepared using a modified protocol for the rapid preparation of DNA from filamentous fungi [34]. The primers ITS 4 (5’-TCCTCCGCTTATTGATATGC-3’) and ITS 5 (5’-GGAAGTAAAAGTCGTAACAAGG-3’) as well as ITS-rDNA amplification conditions were described in our previous report. The resulting ITS-rDNA sequences were edited using the BLASTn program against the NCBI database and were submitted to GenBank to obtain accession numbers.

### Fungal material and preparation EtOAc extracts

The purified fungal isolates were cultured on PDA Petri dishes at 25 °C for 10 days. Then, agar plugs harboring mycelia were obtained using a cork borer, and 2–3 plugs were inoculated into 50-mL Erlenmeyer flasks containing 20 mL of potato dextrose broth (PDB). All flasks were incubated on a rotary shaker at 150 rpm and 28 °C for 7 days to obtain seed cultures. Subsequently, the seed cultures were transferred into 500-mL Erlenmeyer flasks containing 100 g of rice medium under static conditions and incubated at 28 °C for 60 days. The preparation of the rice medium was performed according to the methodology described in our previous report [1].

After incubation, MeOH (3 × 300 mL) was added to each Erlenmeyer flask, followed by maceration and shaking at 100 rpm at room temperature for 72 h, after which each sample was subjected to vacuum filtration. Then, the filtrates were concentrated on a rotary evaporator under reduced pressure to obtain methanolic extracts. The methanolic extracts were then extracted with EtOAc (3 × 300 mL) by partitioning in a separatory funnel (solvent-solvent extraction). Finally, the culture filtrates were concentrated on a rotary evaporator under reduced pressure to obtain EtOAc extracts.

### Antibacterial activity of EtOAc crude extracts

A TLC-3-(4,5-dimethylthiazol-2-yl)-2,5-diphenyl tetrazolium bromide (MTT)-bioautography assay of the samples was performed according to a previously described method [53]. Three gram-positive (*Staphylococcus aureus* ATCC 6538, *Staphylococcus haemolyticus* ATCC 29,970, and *Bacillus subtilis* ATCC 11,562) and four gram-negative (*Escherichia coli* ATCC 25,922, *Pseudomonas lachrymans* ATCC11921, *Xanthomonas vesicatoria* ATCC 11,633, and *Ralstonia solanacearum* ATCC 11,696) bacterial strains were selected for the antibacterial assay. Each EtOAc extract (20 mg) was dissolved in 1 mL of methanol with ultrasonic assistance, after which 5 µL of the sample solution was sampled onto a TLC plate, and TLC was performed using a chloroform (CHCl_3_)-MeOH (15:1, v/v) solvent system. After completion of TLC, 5 µL of streptomycin sulfate (CK^+^) solution (0.2 mg/mL) was sampled onto the lower right side of the TLC plate. Then, the TLC plate was placed on an ultraclean table and ventilated to remove the solvent. The TLC plate was then covered with a bacterial test suspension and incubated at 28 °C for 12 h, after which it was sprayed with the colorimetric reagent MTT (0.5 mg/mL) and incubated for another 2 h. Antibacterial activity was determined by the formation of well-defined inhibition zones made visible by spraying with MTT, which is converted to a formazan dye by living microorganisms. Antibacterial activity was detected as white inhibition zones against a purple background, and the diameter of each antibacterial area was measured. All tests were performed in triplicate.

### Fermentation and extraction of endophytic fungus Eef-7

The fermentation and extraction of secondary metabolites produced by the endophytic fungus Eef-7 were performed according to a previously described method [7]. The endophytic fungus Eef-7 was cultured on PDA medium at 28 °C for 14 days. Then, three agar plugs (0.5 × 0.5 cm) were inoculated into a 500-mL Erlenmeyer flask containing 200 mL of PDB (potato 200 g/L and dextrose 20 g/L) medium and incubated on a rotary shaker at 150 rpm and 28 °C for 7 days. Subsequently, the obtained seed cultures were added to sterilized rice medium (3 kg in total) and incubated at 28 °C for 60 days. The fermented rice material was then extracted with MeOH 3 times, after which the MeOH extracts were dissolved in 1 L of sterilized distilled water and successively partitioned with PE and EtOAc at room temperature. The pooled PE and EtOAc extracts were dried under vacuum to obtain crude extracts of 20.03 g (PE) and 26.15 g (EtOAc).

### Isolation of secondary metabolites produced by endophytic fungus Eef-7

The PE extract (20.03 g) was subjected to column chromatography over a silica gel (200 − 300 mesh) eluted with a gradient of PE-acetone (100:0–0:100) to afford eleven fractions. Fraction 9 (256 mg) was chromatographed over Sephadex LH-20 (eluted with CHCl_3_-MeOH, 1:1) to obtain six subfractions. Subfraction 3 (164.2 mg) was further purified by semi-preparative HPLC elution with a gradient of MeOH-H_2_O (0–5 min, MeOH 40 %; 5–45 min, MeOH 40-100 %; flow rate 4 mL/min with an isocratic 0.01 % TFA modifier) to obtain compound 2. Fraction 11 (122 mg) was also chromatographed over Sephadex LH-20 (eluted with CHCl_3_-MeOH, 1:1) to obtain four subfractions. Subfraction 2 (16 mg) was further purified by semi-preparative HPLC eluting with MeOH-H_2_O (70:30, flow rate 4 mL/min with an isocratic 0.01 % TFA modifier) to obtain compound 1.

The EtOAc extract (26.15 g) was subjected to column chromatography over silica gel (200–300 mesh) eluted with a gradient of PE-acetone (100:0–0:100) to obtain fourteen fractions. Fraction 5 (140 mg) was chromatographed over Sephadex LH-20 (eluted with CHCl_3_-MeOH, 1:1) to obtain six subfractions. Subfraction 2 (68 mg) was further purified by semi-preparative HPLC eluting with MeOH-H_2_O (25:75, with an isocratic 0.01 % TFA modifier, 4 mL/min) to afford compound 3. Fractions 6–8 (398 mg) was also chromatographed over Sephadex LH-20 (eluted with CHCl_3_-MeOH, 1:1) to obtain five subfractions. Compound 1 (182.4 mg) was crystallized from subfractions 2–4 and further purified by recrystallization.

### Antibacterial assay of pure compounds isolated from the endophytic fungus Eef-7

*R. solanacearum* was used to assess the antibacterial activity of pure compounds. Streptomycin sulfate was used as a positive control. The inhibition zone diameters of the compounds and positive control were determined using the modified filter paper diffusion method as previously described [54].

## Supplementary Information

**Additional file 1:Sup Fig. S1** HR-ESI-MS spectrum of scorpinone (1). **Sup Fig. S2**^1^ H NMR spectrum of scorpinone (1) (CDCl_3_, 600 MHz). **Sup Fig. S3**^13^ C NMR spectrum of scorpinone (1) (CDCl_3_, 151 MHz).  **Sup Fig. S4**^1^-^1^ H COSY spectrum of scorpinone (1) (CDCl_3_, 151 MHz). **Sup Fig. S5** HSQC spectrum of scorpinone (1) (CDCl_3_, 151 MHz). **Sup Fig. S6** HMBC spectrum of scorpinone (1) (CDCl_3_, 151 MHz). **Sup Fig. S7** HR-ESI-MS spectrum of 5-deoxybostrycoidin (2). **Sup Fig. S8**^1^ H NMR spectrum of 5-deoxybostrycoidin (2) (CDCl_3_, 600 MHz). **Sup Fig. S9**^13^ C NMR spectrum of 5-deoxybostrycoidin (2) (CDCl_3_, 151 MHz). **Sup Fig. S10**^1^-^1^ H COSY spectrum of 5-deoxybostrycoidin (2) (CDCl_3_, 151 MHz). **Sup Fig. S11** HSQC spectrum of 5-deoxybostrycoidin (2) (CDCl_3_, 151 MHz). **Sup Fig. S12** HMBC spectrum of 5-deoxybostrycoidin (2) (CDCl_3_, 151 MHz). **Sup Fig. S13**^1^ H NMR spectrum of 4-methyl-5,6-dihydro-2-pyranone (3) (acetone-*d*_6_, 600 MHz). **Sup Fig. S14**^13^ C NMR spectrum of 4-methyl-5,6-dihydro-2-pyranone (3) (acetone-*d*_6_, 151 MHz).

## Data Availability

The generated nucleotide sequence of the endophytic fungal isolates (isolation number Eef-1~Eef-15) can be accessed in GenBank under accession numbers MK120854 to MK120868(https://blast.ncbi.nlm.nih.gov/Blast.cgi). The datasets generated and/or analyzed during the current study are available from the corresponding author on reasonable request.
