# Supplementary material for: Diversity and antibacterial activity of fungal endophytes from Eucalyptus exserta
Source: BMC Microbiol. 2021 May 27;21:155. doi: 10.1186/s12866-021-02229-8 (PMC8157698; doi:10.1186/s12866-021-02229-8)
Supplement: Supplementary file 1 — Additional file 1:Sup Fig. S1 HR-ESI-MS spectrum of scorpinone (1). Sup Fig. S21 H NMR spectrum of scorpinone (1) (CDCl3, 600 MHz). Sup Fig. S313 C NMR spectrum of scorpinone (1) (CDCl3, 151 MHz). Sup Fig. S41-1 H COSY spectrum of scorpinone (1) (CDCl3, 151 MHz). Sup Fig. S5 HSQC spectrum of scorpinone (1) (CDCl3, 151 MHz). Sup Fig. S6 HMBC spectrum of scorpinone (1) (CDCl3, 151 MHz). Sup Fig. S7 HR-ESI-MS spectrum of 5-deoxybostrycoidin (2). Sup Fig. S81 H NMR spectrum of 5-deoxybostrycoidin (2) (CDCl3, 600 MHz). Sup Fig. S913 C NMR spectrum of 5-deoxybostrycoidin (2) (CDCl3, 151 MHz). Sup Fig. S101-1 H COSY spectrum of 5-deoxybostrycoidin (2) (CDCl3, 151 MHz). Sup Fig. S11 HSQC spectrum of 5-deoxybostrycoidin (2) (CDCl3, 151 MHz). Sup Fig. S12 HMBC spectrum of 5-deoxybostrycoidin (2) (CDCl3, 151 MHz). Sup Fig. S131 H NMR spectrum of 4-methyl-5,6-dihydro-2-pyranone (3) (acetone-d6, 600 MHz). Sup Fig. S1413 C NMR spectrum of 4-methyl-5,6-dihydro-2-pyranone (3) (acetone-d6, 151 MHz). [file 12866_2021_2229_MOESM1_ESM.docx]

Supporting Information

**Diversity and antibacterial activities of fungal endophytes from *Eucalyptus exserta***

Ziling Mao^1^, Weihao Zhang^1^, Chunyin Wu^1^, Hao Feng^1^, Yuanhang Peng^1^, Hamza Shahid^1^, Zining Cui^2^, Ping Ding^3^*, Tijiang Shan^1^*

^1^ Guangdong Key Laboratory for Innovative Development and Utilization of Forest Plant Germplasm, College of Forestry and Landscape Architecture, South China Agricultural University, Guangzhou, 510642, China

^2^ Guangdong Province Key Laboratory of Microbial Signals and Disease Control, South China Agricultural University, Guangzhou, 510642, China

^3^ School of Pharmaceutical Sciences, Guangzhou University of Chinese Medicine, Guangzhou 510006, China

Corresponding Author:

Ping Ding

No. 232, Waihuandong Road, Panyu District, Guangzhou, Guangdong, 510006, China

Email address: dingpinggz@126.com

Tijiang Shan

No. 483, Wushan Road, Tianhe District, Guangzhou, Guangdong, 510642, China

Email address: tjshan@scau.edu.cn


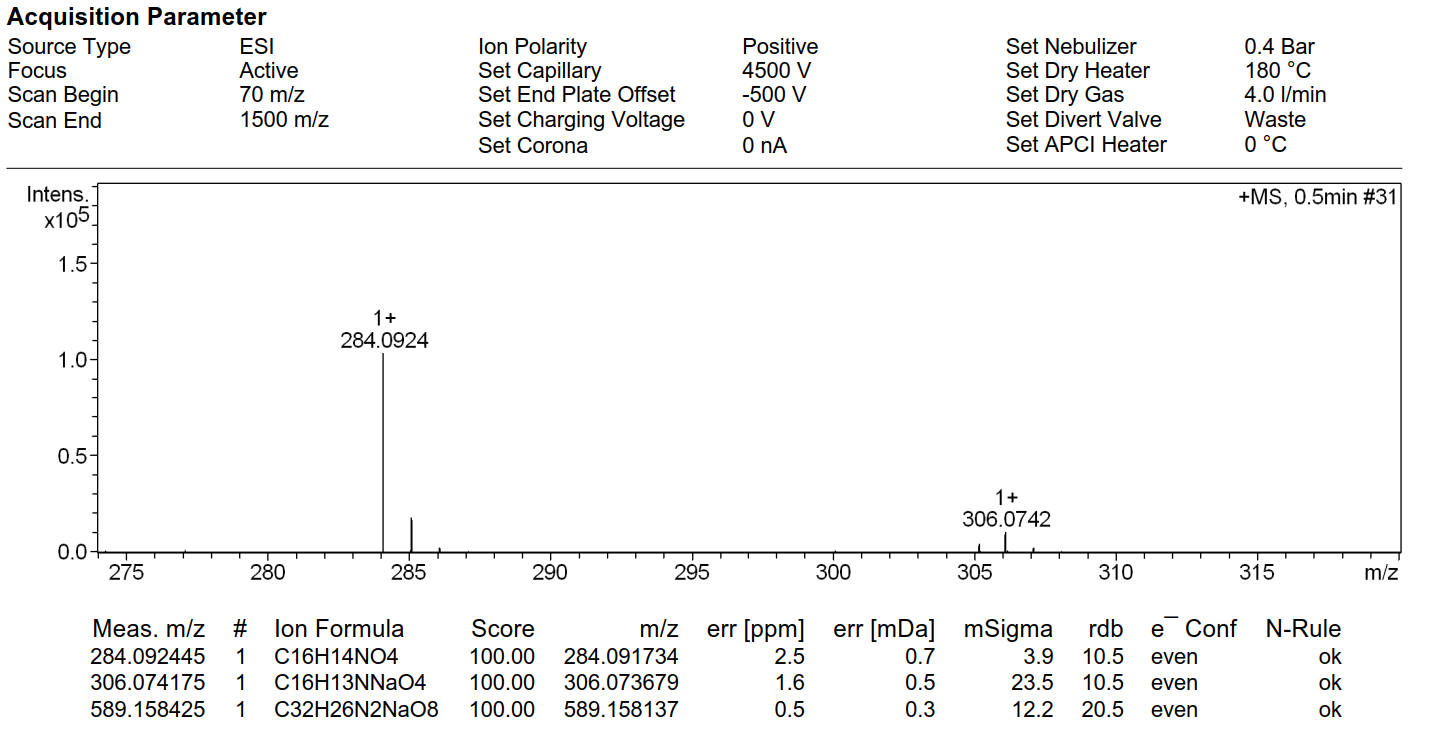


# Fig. S1 HR-ESI-MS spectrum of scorpinone (1)


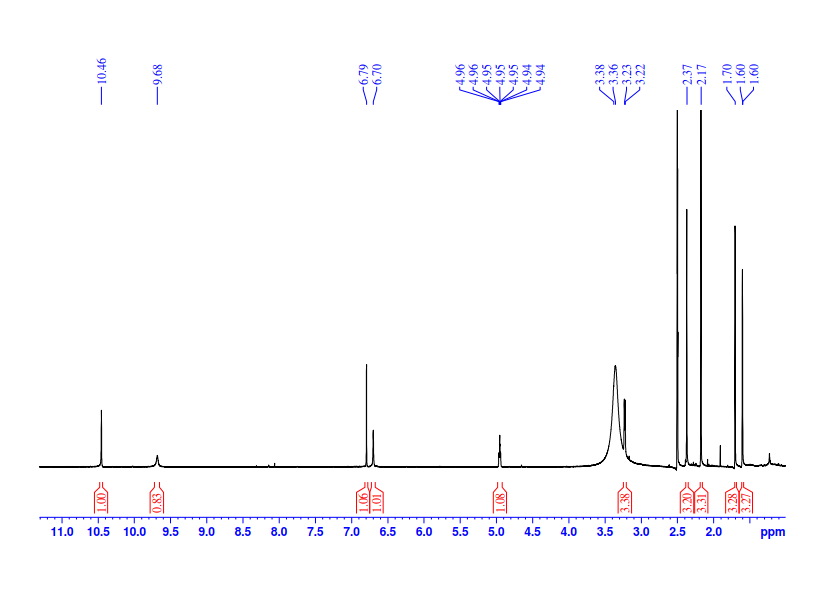


# Fig. S2 ^1^H NMR spectrum of scorpinone (1) (CDCl_3_, 600 MHz)


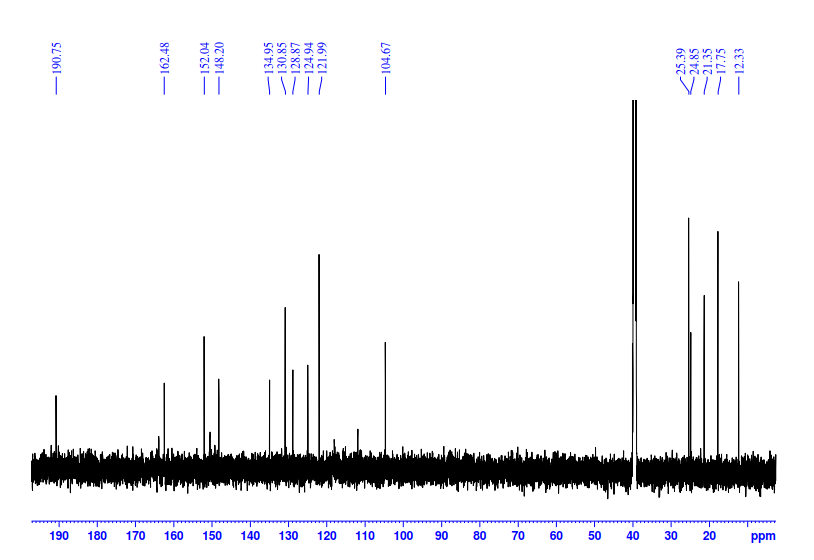


# Fig. S3 ^13^C NMR spectrum of scorpinone (1) (CDCl_3_, 151 MHz)


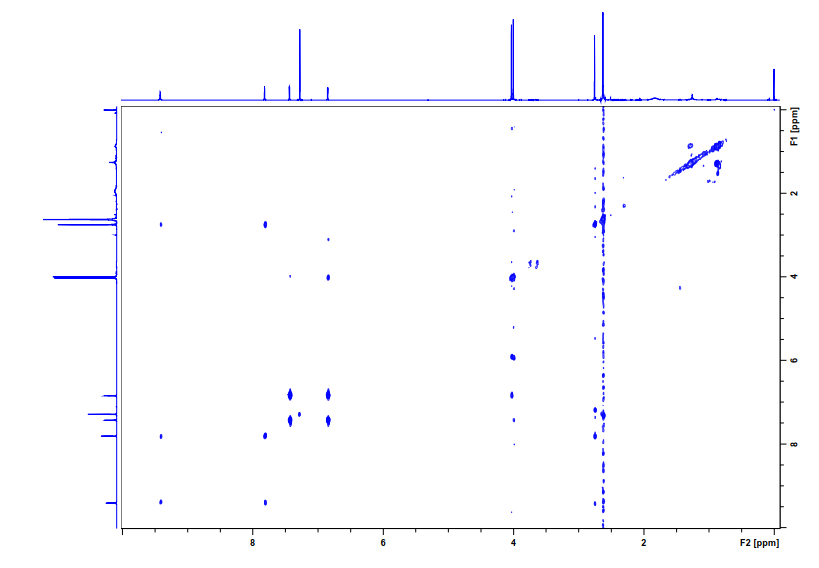
**Fig. S4** ^1^H-^1^H COSY spectrum of scorpinone (1) (CDCl_3_, 151 MHz)


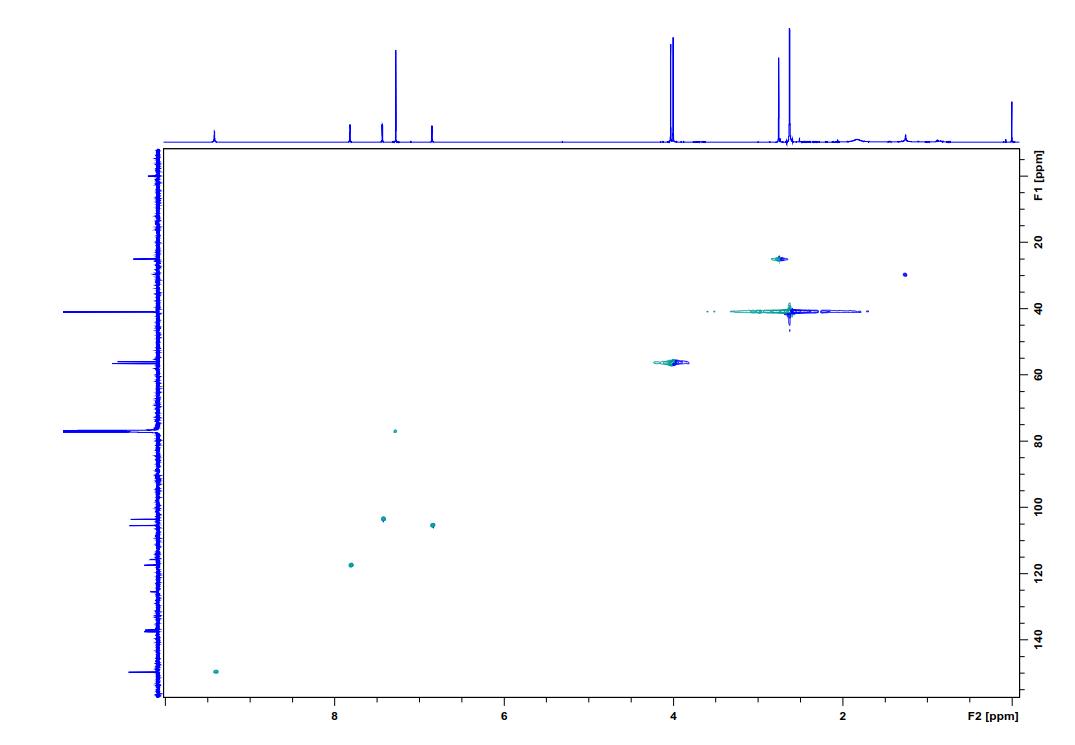


# Fig. S5 HSQC spectrum of scorpinone (1) (CDCl_3_, 151 MHz)


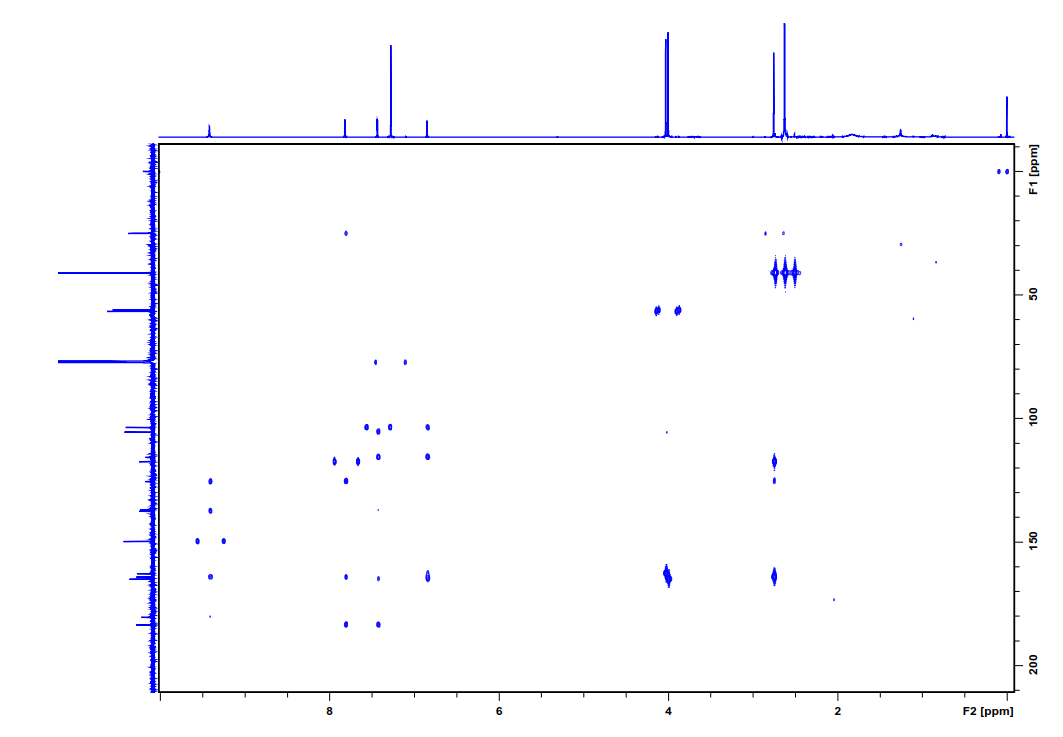


# Fig. S6 HMBC spectrum of scorpinone (1) (CDCl_3_, 151 MHz)


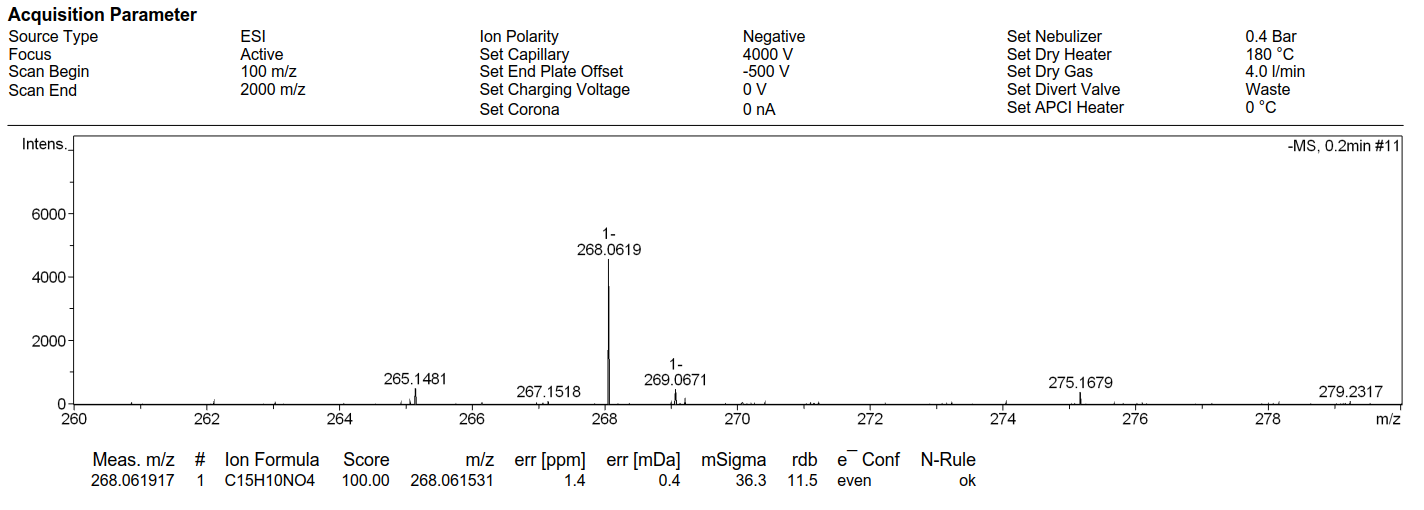


# Fig. S7 HR-ESI-MS spectrum of 5-deoxybostrycoidin (2)


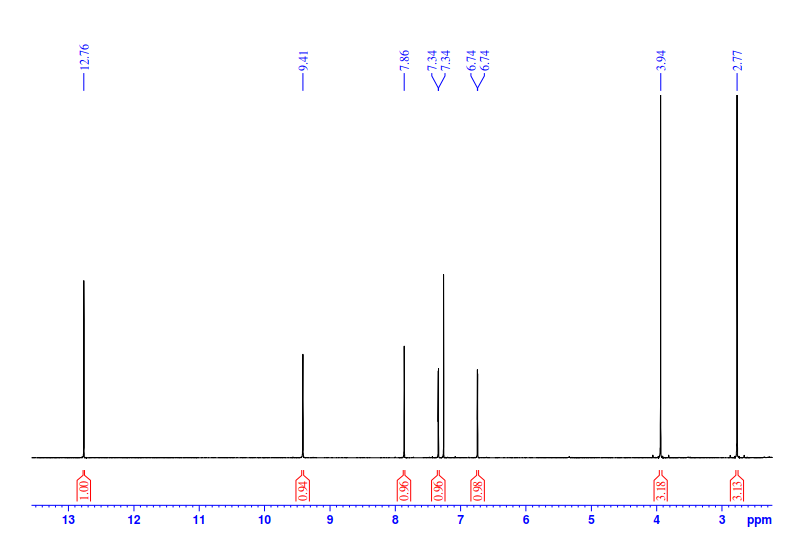


# Fig. S8 ^1^H NMR spectrum of 5-deoxybostrycoidin (2) (CDCl_3_, 600 MHz)


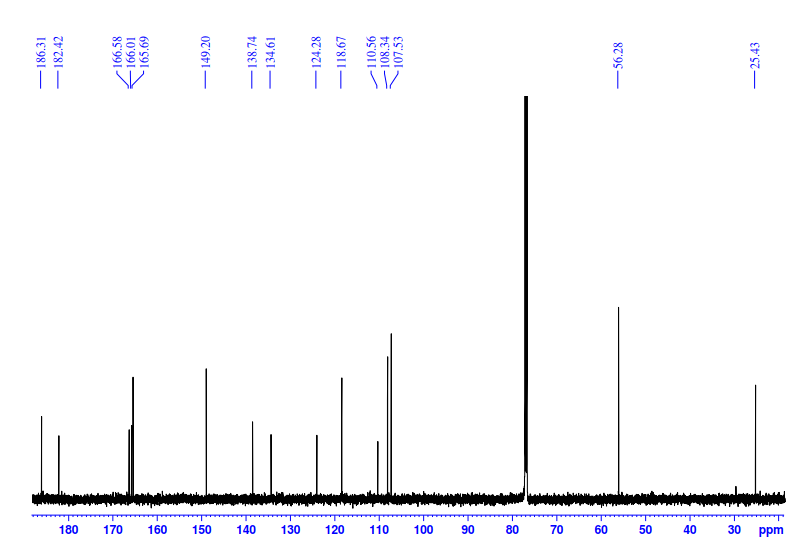


# Fig. S9 ^13^C NMR spectrum of 5-deoxybostrycoidin (2) (CDCl_3_, 151 MHz)


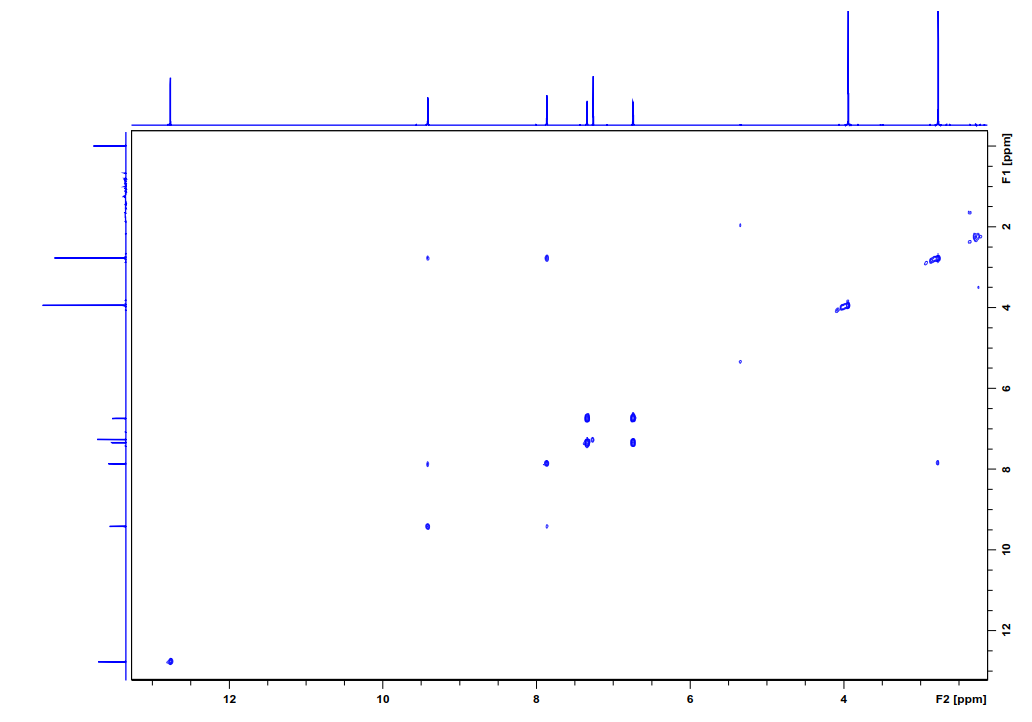


# Fig. S10 ^1^H-^1^H COSY spectrum of 5-deoxybostrycoidin (2) (CDCl_3_, 151 MHz)


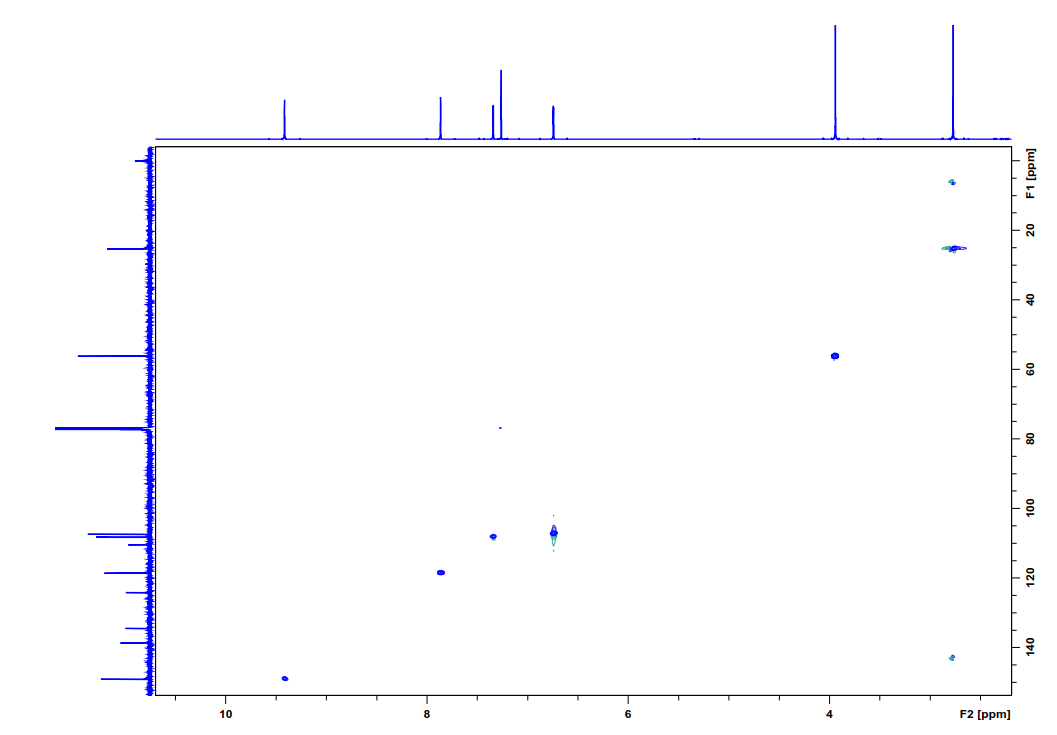


# Fig. S11 HSQC spectrum of 5-deoxybostrycoidin (2) (CDCl_3_, 151 MHz)


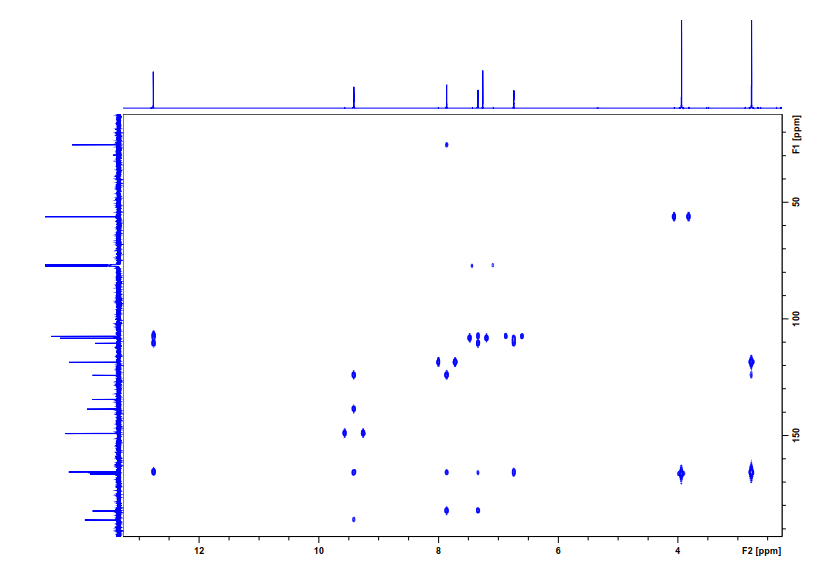


# Fig. S12 HMBC spectrum of 5-deoxybostrycoidin (2) (CDCl_3_, 151 MHz)


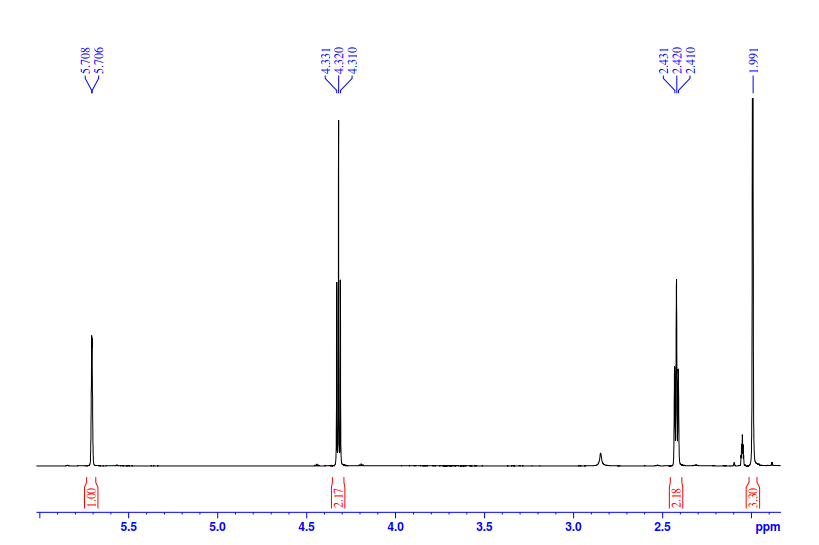


# Fig. S13 ^1^H NMR spectrum of 4-methyl-5, 6-dihydro-2-pyranone (3) (Acetone-*d*_6_, 600 MHz)


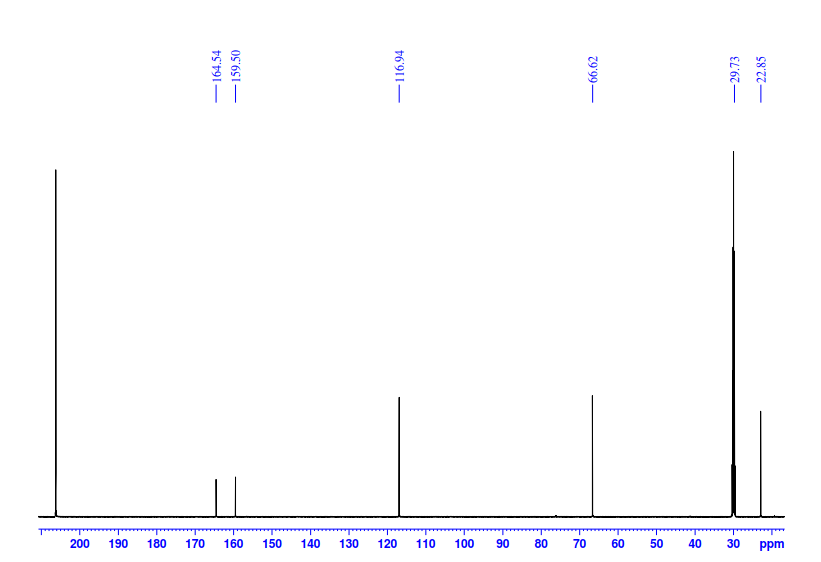


# Fig. S14 ^13^C NMR spectrum of 4-methyl-5, 6-dihydro-2-pyranone (3) (Acetone-*d*_6_, 151 MHz)
